# Supplementary material for: Transcriptomic Insights into Mechanisms of Early Seed Maturation in the Garden Pea (Pisum sativum L.)
Source: Cells. 2020 Mar 23;9(3):779. doi: 10.3390/cells9030779 (PMC7140803; doi:10.3390/cells9030779)
Supplement: Supplementary file 1 [file cells-09-00779-s001.zip › Supplementary materials/FileS2.zip!/FileS1/Summary Sheets - Modules/Overexpression Spots/Report.pdf]

Overexpression Spots

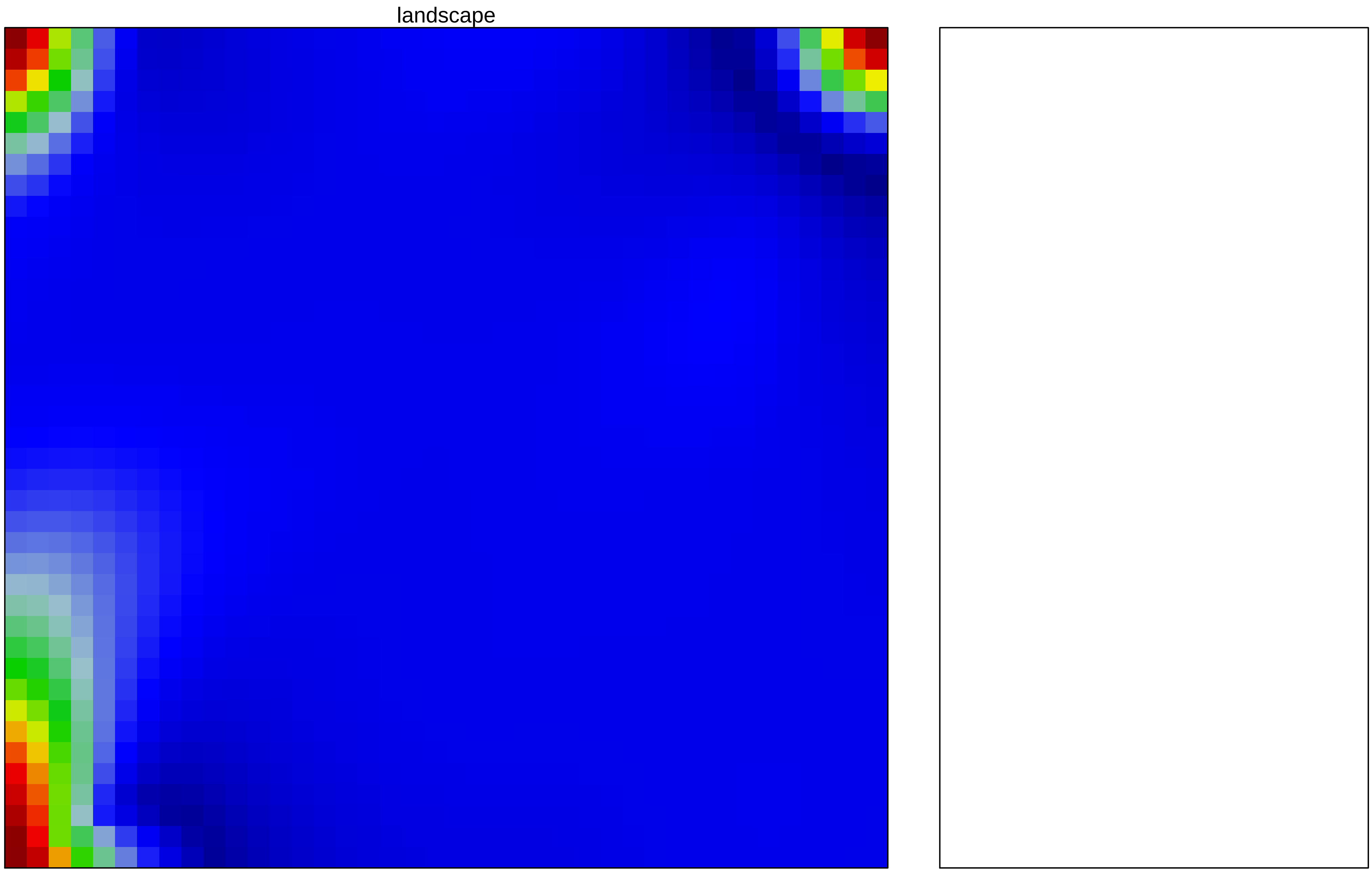

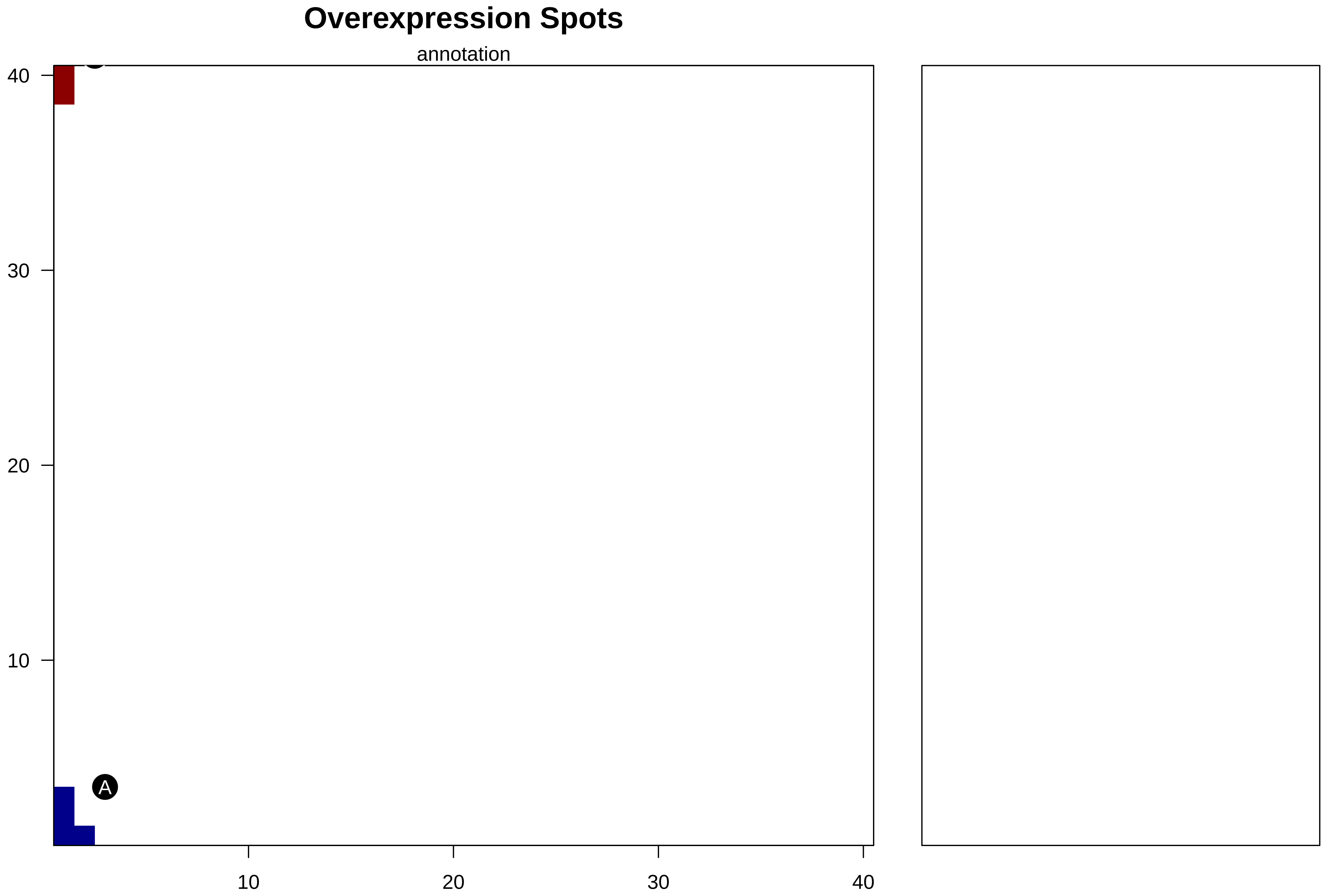

Overexpression Spots
annotation
40
30
20
10
A
10
20
30
40

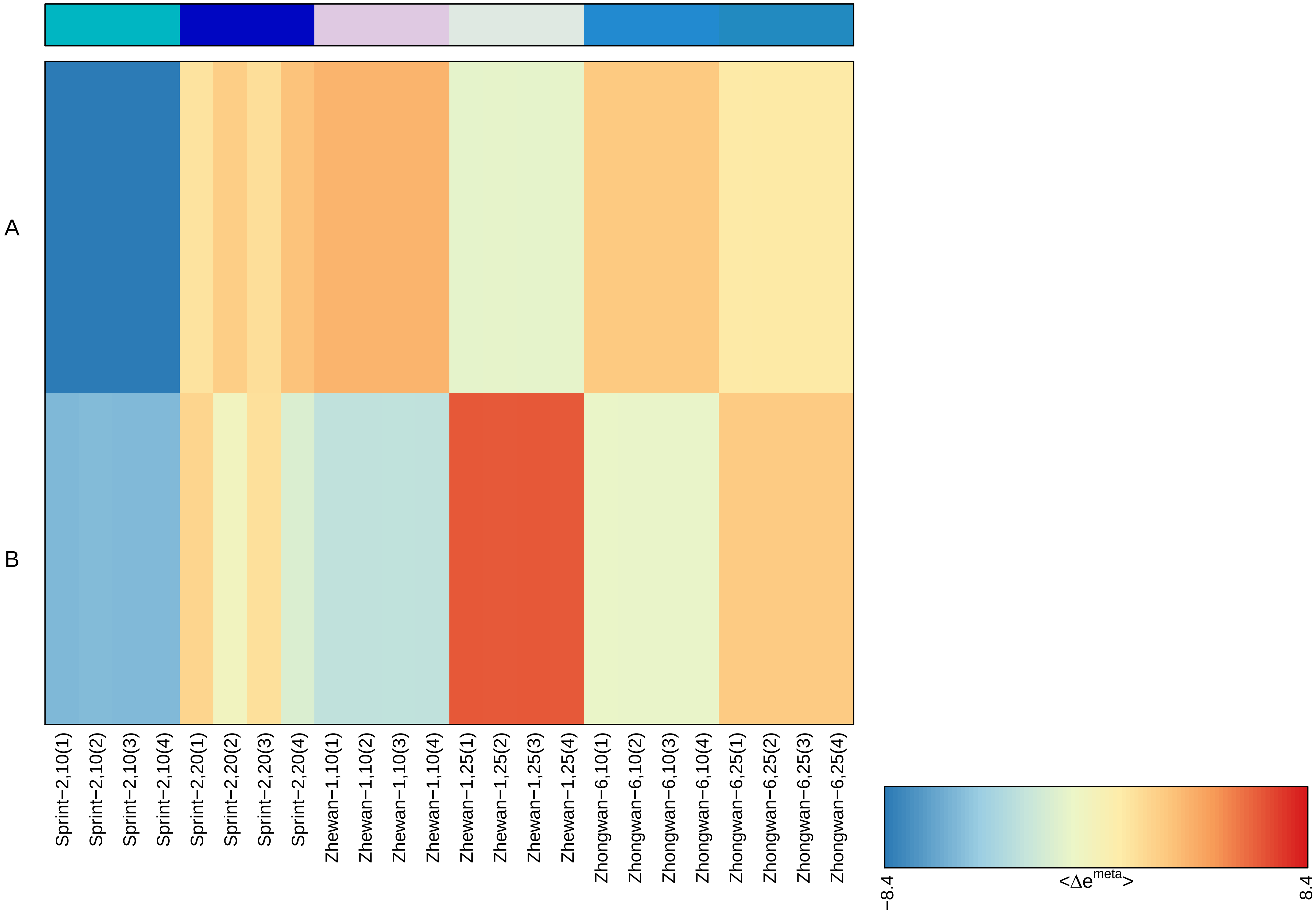

A
B
Sprint−2,10(1)
Sprint−2,10(2)
Sprint−2,10(3)
Sprint−2,10(4)
Sprint−2,20(1)
Sprint−2,20(2)
Sprint−2,20(3)
Sprint−2,20(4)
Zhewan−1,10(1)
Zhewan−1,10(2)
Zhewan−1,10(3)
Zhewan−1,10(4)
Zhewan−1,25(1)
Zhewan−1,25(2)
Zhewan−1,25(3)
Zhewan−1,25(4)
Zhongwan−6,10(1)
Zhongwan−6,10(2)
Zhongwan−6,10(3)
Zhongwan−6,10(4)
Zhongwan−6,25(1)
Zhongwan−6,25(2)
Zhongwan−6,25(3)
Zhongwan−6,25(4)
−8.4
<Δe^meta>
8.4

Overexpression Spots

Spot Summary: A

metagenes = 4
genes = 38

<r> metagenes = 1
<r> genes = 0.63
beta: r2= 25.35 / log p= −Inf

samples with spot = 0 (0 %)

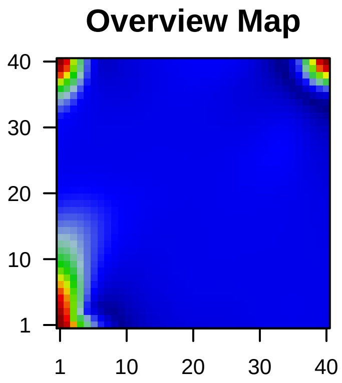

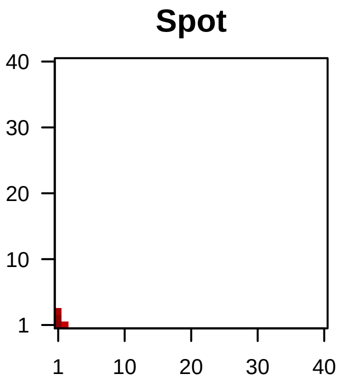

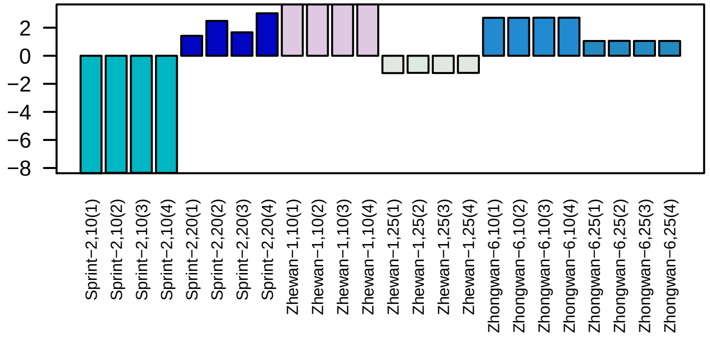

Spot Genelist

Rank	ID	max e	min e	r	Symbol	Description
1	TRINITY_DN2	23.2	−19.57	0.8		
2	TRINITY_DN1	21.33	−106.92	0.92		
3	TRINITY_DN7	20.26	−13.18	0.8		
4	TRINITY_DN4	19.94	−13.52	0.56		
5	TRINITY_DN3	16.43	−9.68	0.65		
6	TRINITY_DN1	13.69	−6.37	0.68		
7	TRINITY_DN7	13.41	−12.69	0.9		
8	TRINITY_DN3	13.38	−29.37	0.9		
9	TRINITY_DN2	11.58	−8.49	0.75		
10	TRINITY_DN3	10.97	−5.32	0.58		
11	TRINITY_DN3	9.96	−9.05	0.55		
12	TRINITY_DN7	7.7	−5.96	0.71		
13	TRINITY_DN3	5.8	−7.86	0.77		
14	TRINITY_DN3	5.67	−6.93	0.79		
15	TRINITY_DN3	5.25	−4.21	0.68		
16	TRINITY_DN3	4.83	−3.23	0.71		
17	TRINITY_DN3	4.13	−3.93	0.79		
18	TRINITY_DN1	4.04	−4.03	0.75		
19	TRINITY_DN6	3.63	−3.54	0.87		
20	TRINITY_DN1	3.44	−2.28	0.77		

Overexpression Spots

Spot Summary: B

metagenes = 2
genes = 16

<r> metagenes = 1
<r> genes = 0.83
beta: r2= 10.02 / log p= −Inf

samples with spot = 4 (16.7 %)
Zhewan−1(25) : 4 (100 %)

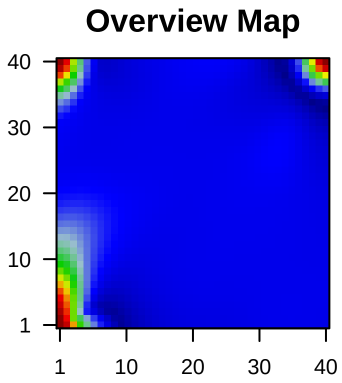

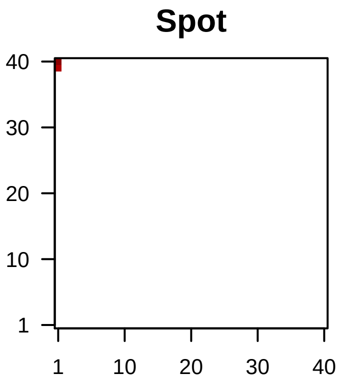

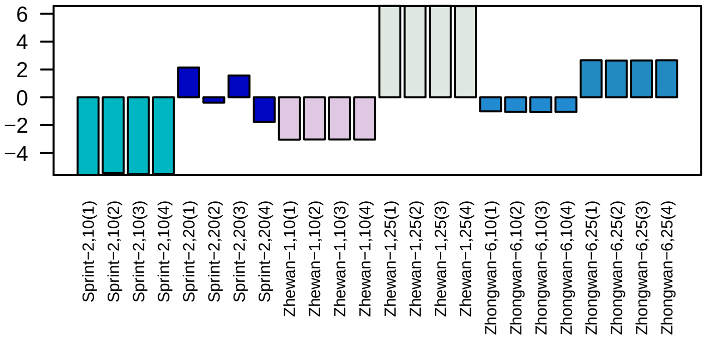

Spot Genelist

Rank	ID	max e	min e	r	Symbol	Description
1	TRINITY_DN2	19.02	−23.73	0.94		
2	TRINITY_DN6	13.45	−12.66	0.97		
3	TRINITY_DN2	10.34	−9.73	0.93		
4	TRINITY_DN4	10.09	−9.97	0.95		
5	TRINITY_DN4	9.16	−4.51	0.88		
6	TRINITY_DN2	8.02	−4.57	0.98		
7	TRINITY_DN2	7.51	−8.63	0.93		
8	TRINITY_DN4	6.31	−3.15	0.94		
9	comp39373_c	6.03	−3.43	0.98		
10	TRINITY_DN2	3.51	−1.01	0.86		
11	TRINITY_DN3	2.68	−3.01	0.62		
12	TRINITY_DN1	2.46	−0.77	0.75		
13	TRINITY_DN3	2.45	−1.45	0.98		
14	TRINITY_DN1	2.42	−1.79	0.84		
15	TRINITY_DN3	1.73	−0.78	0.89		
16	TRINITY_DN2	1.69	−1.29	0.89		
